# Laser Vibrometry and Dispersion Analysis-Enabled Mechanical Property Characterization for Additively Manufactured Thermoset Composites

**DOI:** 10.34133/research.1455

**Published:** 2026-10-19

**Authors:** Bowen Cai, Andrew Moomaw, Yingshan Du, Teng Li, Jiali Li, Luyu Bo, Rani Sullivan, Wayne Huberty, Chuangchuang Sun, Zhenhua Tian

**Affiliations:** ^1^ Department of Aerospace Engineering, Mississippi State University, Mississippi State, MS, USA.; ^2^ Advanced Composites Institute, Mississippi State University, Starkville, MS, USA.; ^3^ Department of Mechanical Engineering, Virginia Polytechnic Institute and State University, Blacksburg, VA, USA.; ^4^ Department of Biomedical Engineering, Virginia Polytechnic Institute and State University, Blacksburg, VA, USA.; ^5^ Department of Mechanical Engineering, Villanova University, Villanova, PA, USA.

## Abstract

Large-format additive manufacturing of thermoset composites enables rapid fabrication of large structures, but nondestructive characterization of their print-path-dependent mechanical behavior remains difficult. Existing qualification routes rely mainly on destructive coupon testing or defect-oriented nondestructive evaluation and therefore provide limited capability for targeted mapping of direction-dependent mechanical variability. This study presents a piezoelectric transducer–laser Doppler vibrometry framework for direction-conditioned dispersion inversion of full-field guided wave measurements in additively manufactured thermoset composites. The method decomposes the frequency–wavenumber spectrum by propagation angle and independently fits the extracted dispersion data in each propagation direction to obtain scan-region-specific effective shear-wave velocity, an effective Poisson’s-ratio parameter, and effective-modulus responses as functions of propagation direction. Benchmark testing on an isotropic aluminum plate validates that the workflow recovers isotropic guided wave behavior without introducing artificial angular bias. Applied to a chopped-carbon-fiber thermoset composite, the framework reveals print-path-dependent anisotropic wave propagation through elliptical wavefields and frequency–wavenumber spectra. The resulting effective stiffness estimates reproduce the direction-dependent stiffness variation and approximate magnitude observed in conventional tensile measurements, supporting the mechanical relevance of the guided wave-derived response. By converting full-field guided wave measurements into scan-region-specific, direction-resolved effective-stiffness estimates, this work provides a practical nondestructive route to support quality control, process validation, and structural evaluation of aerospace composite tooling manufactured by large-format thermoset additive manufacturing.

## Introduction

Large-format additive manufacturing (LFAM) enables meter-scale structures to be produced within days or even hours and has become increasingly attractive for aerospace composite manufacturing because it can reduce material waste, labor cost, and delivery time [1–4]. In particular, additive manufacturing can integrate features such as vacuum channels, conformal heating and cooling circuits, and geometric references directly into composite layup molds, thereby simplifying downstream manufacturing processes [5]. For such tooling and structural applications, the matrix system is critical because it governs bonding, thermal stability, and dimensional integrity under processing and service conditions. Although thermoplastic LFAM systems have been widely explored, their weak through-thickness bonding, high thermal demands during processing, and thermal softening can limit their structural applications in high-temperature tooling environments [6]. In contrast, thermoset-based LFAM platforms offer stronger through-thickness bonding after curing and improved thermal stability, which has driven growing interest in technologies such as reactive additive manufacturing (RAM) and continuous-fiber 3-dimensional printing (CF3D) [7–9]. However, because LFAM produces large tooling structures through layer-by-layer deposition, process-induced anisotropy, incomplete curing, porosity, and local inhomogeneity can become harder to control, detect, and qualify as build size increases. For aerospace composite tooling, this print-path-dependent variability makes reliable assessment of direction-dependent stiffness response a critical qualification issue. Although existing qualification approaches can provide useful material-property or defect information, they do not readily provide a nondestructive way to quantify how stiffness varies with direction across large printed tooling.

For anisotropic media, especially additively manufactured structures, mechanical-property characterization is highly challenging. Destructive coupon testing can provide direction-specific tensile properties, but it samples only selected extracted directions and is not practical for dense spatial coverage of large printed structures. Bulk-wave ultrasonic methods can identify useful orthotropic material constants, but they typically require controlled specimen geometries, known propagation paths, and predefined material symmetry assumptions [10,11]. Defect and geometry-oriented nondestructive evaluation (NDE) methods, such as x-ray computed tomography (CT) and micro-CT, are valuable for assessing porosity, internal defects, and dimensional fidelity in additively manufactured structures, but they are not primarily intended to produce direction-resolved effective stiffness-response maps within selected inspection regions [12–18]. Therefore, there is a strong need for NDE methods capable of quantifying directional variations in stiffness-related material behavior to ensure structural integrity and reliable performance [19].

Among available NDE approaches, ultrasonic guided waves are particularly attractive for this problem. Guided waves can travel long distances in plate-like structures with relatively low energy loss, making them suitable for inspecting large areas [20–24]. In addition, they are highly sensitive to material discontinuities and internal damage, because defects such as cracks, delamination, corrosion, and weak interfaces alter the wave propagation through scattering, mode conversion, attenuation, and velocity changes. More importantly, such waves are dispersive and multimodal [25,26]. Their frequency–wavenumber relations are strongly influenced by material properties and geometric parameters, which provides a pathway for inferring mechanical properties from measured wave propagation behavior [27–30]. For these reasons, guided wave-based inspection has become an effective tool for metallic, composite, and additively manufactured structures. Building on this established capability, the present challenge for LFAM thermoset composite tooling is to translate measured direction-dependent guided wave dispersion into effective stiffness estimates that can be assessed against mechanical measurements.

Achieving this conversion requires wavefield information that is sufficiently dense and directional for anisotropic materials [31]. In conventional guided wave testing, a sparse set of transducers is commonly arranged to monitor signals along a small number of fixed paths. Such sparse measurements can be effective in isotropic or quasi-isotropic structures [32,33], but they are insufficient when the material response varies continuously with propagation direction. Additively manufactured thermosets can exhibit anisotropy and inhomogeneity associated with the deposition path, possible fiber-orientation bias, inter-bead interfaces, porosity, and local defects [13,14,34]. Under these conditions, wave behavior along a few selected paths does not provide a complete description of the material response. In addition, installing numerous contact sensors and wires may introduce unwanted mass loading and measurement uncertainties. Therefore, guided wave-based characterization of additively manufactured (AM) thermoset composites requires a noncontact wavefield acquisition approach with high sensitivity and spatial resolution.

Laser Doppler vibrometry (LDV) provides such a noncontact measurement route [35,36]. An LDV can remotely scan the specimen surface and acquire high-sensitivity vibration data without the sensor-loading effects introduced by bonded receivers. More importantly, scanning LDV enables acquisition of the full-field wavefield, or at least a dense set of waveforms over a prescribed region, which is highly advantageous for understanding direction-dependent guided wave behavior. Previous studies have already demonstrated the potential of LDV-based guided wave characterization [37,38]. Ambrozinski et al. [39] used scanning LDV measurements in an aluminum plate together with the multiple signal classification (MUSIC) method to identify Young’s modulus. Kudela et al. [10] acquired and analyzed guided wave fields in carbon fiber-reinforced polymer (CFRP) plates and inverted a complete set of orthotropic elastic constants. Chang and Yuan [40] used one-dimensional LDV scans and the Matrix Pencil method to extract guided wave dispersion curves in isotropic and anisotropic plates. Moll et al. [41] used scanning LDV to investigate the influence of damage on guided wave propagation and dispersion in stiffened carbon-fiber composite plates. Malatesta et al. [42] developed wavefield analysis tools to extract wavenumber-frequency relations of A_0_ waves in anisotropic CFRP plates as a function of propagation direction.

Taken together, these studies establish an important basis for LDV guided wave characterization, including full-field wavefield acquisition, dispersion extraction, and elastic-property inversion in metallic and composite plates. However, these capabilities have generally been pursued toward 2 distinct objectives: Property-inversion studies seek constitutive material-property identification through prescribed material models, whereas direction-resolved wavefield analyses primarily quantify angular wavenumber- or velocity-based propagation characteristics. The gap between direction-resolved propagation analysis and mechanically referenced stiffness estimation is particularly relevant to LFAM thermoset composite tooling, where the deposition path, bead continuity, fiber-orientation bias, inter-bead interfaces, porosity, cure variation, and local defects can introduce both location-dependent and direction-dependent guided wave responses. The qualification need is therefore a method that converts measured directional dispersion into stiffness-related estimates within selected scan regions and links those estimates to direction-dependent tensile stiffness. This study therefore presents a piezoelectric transducer–laser Doppler vibrometry (PZT-LDV) framework for direction-conditioned dispersion inversion that converts dense wavefield measurements into scan-region-specific directional effective properties and evaluates those properties against ASTM D638 tensile measurements and a tensile-constrained in-plane orthotropic stiffness trend.

In the proposed PZT-LDV guided wave framework, a single bonded PZT actuator excites broadband guided waves, and a scanning LDV measures the out-of-plane response over a dense spatial grid to assemble the time–space wavefield. Directional frequency–wavenumber slicing decomposes the frequency–wavenumber spectrum $U\left(f,k_{x},k_{y}\right)$ into propagation-angle-specific dispersion profiles $V\left(\theta ,f,k\right).$ Mode-labeled A_0_ and conditionally retained S_0_ dispersion points from each profile are then fitted against Rayleigh–Lamb dispersion curves to estimate the effective shear-wave velocity $c_{s,\mathrm{eff}}\left(\theta \right)$ and an effective Poisson’s-ratio parameter $\nu_{\mathrm{eff}}\left(\theta \right),$ which characterize the direction-dependent guided wave response. These fitted quantities are converted into a guided wave-derived effective modulus $E_{\mathrm{eff}}\left(\theta \right)$ for stiffness-trend comparison. By conditioning the regression on θ, the measured dispersion in each propagation direction is evaluated independently to obtain direction-resolved effective-property estimates. The workflow is verified on an isotropic aluminum benchmark, applied to an additively manufactured chopped-carbon-fiber thermoset composite, and compared with ASTM D638 tensile measurements and a tensile-constrained in-plane orthotropic surrogate. The resulting direction-wise estimates are subsequently assessed against the tensile measurements, establishing an experimental connection between measured directional guided wave dispersion and direction-dependent effective stiffness for nondestructive characterization of LFAM thermoset composite tooling.

## Results

### PZT-LDV guided wave sensing and analysis framework

To characterize direction-dependent effective mechanical properties in large-format additively manufactured thermoset composites, the proposed framework treats the scanned guided wavefield as a quantitative link between print-path architecture and material-property estimation, rather than merely as a visualization of wave propagation. Figure 1 schematizes this sensing-to-property route through 3 connected modules: fabrication of the print-path-dependent thermoset composite, PZT-LDV full-field wavefield acquisition and frequency–wavenumber processing, and directional effective-property estimation.

**Fig. 1.**
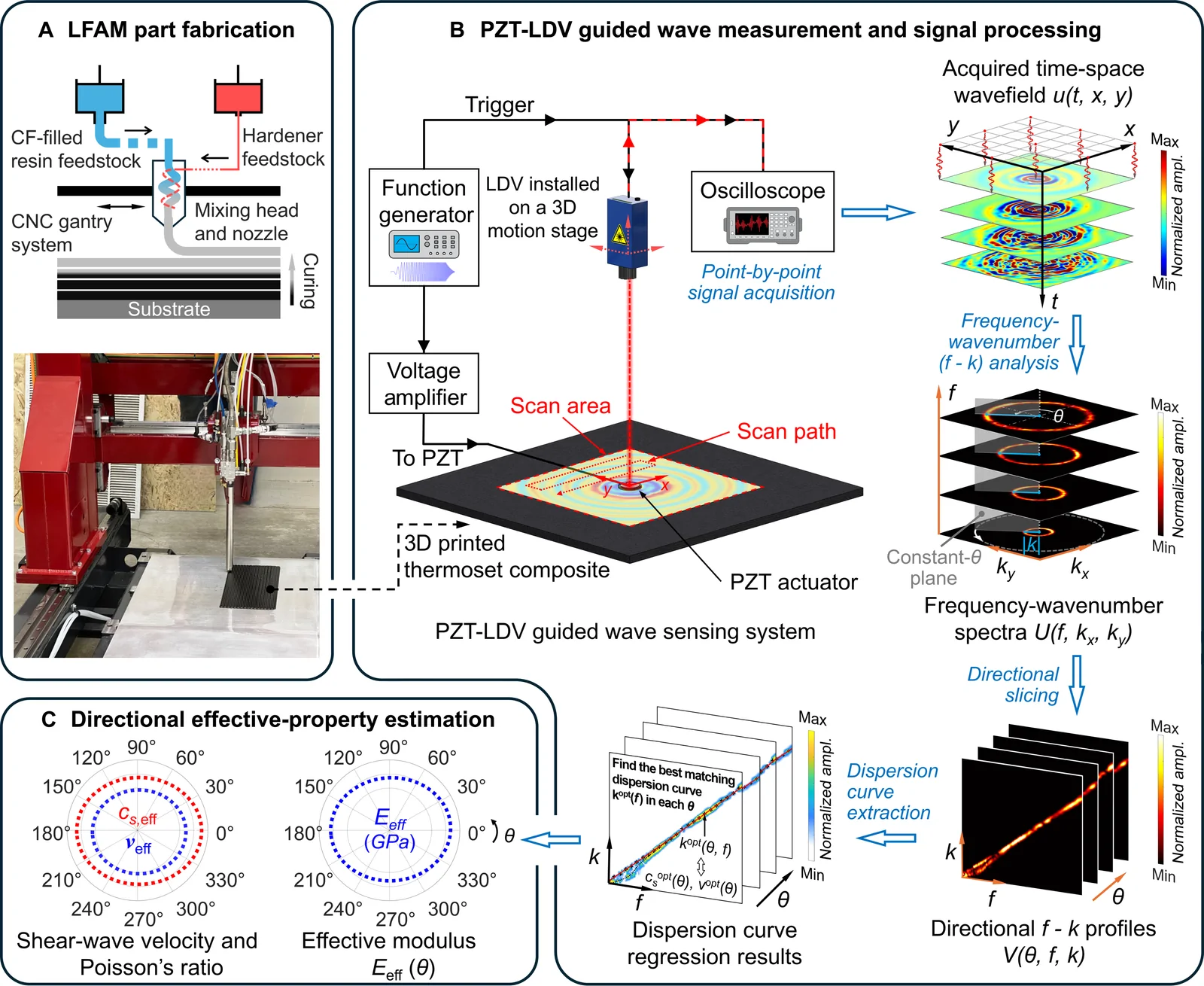
PZT-LDV wavefield-to-property workflow for direction-resolved effective-property characterization of additively manufactured thermoset composites. (A) LFAM thermoset composite fabrication and print-path-dependent architecture. (B) Single-PZT guided wave excitation, scanning-LDV wavefield acquisition, frequency–wavenumber transformation, and directional slicing. (C) Directional dispersion curve regression and effective-property estimation.

Figure 1A illustrates the LFAM fabrication process and the resulting print-path architecture, with the print-path direction used as the angular reference for the subsequent guided wave analysis. In Fig. 1B, a bonded PZT actuator excites broadband guided waves, while scanning LDV acquires the full-field out-of-plane response; the measured wavefield is transformed into the frequency–wavenumber domain and resolved by propagation direction to obtain directional dispersion information. In Fig. 1C, the directional dispersion data are analyzed through direction-wise regression to estimate the effective shear-wave velocity $c_{s,\mathrm{eff}\;}\left(\theta \right)$ and effective Poisson’s-ratio parameter $\nu_{\mathrm{eff}}\;\left(\theta \right)$, from which the guided wave-derived effective modulus $E_{\mathrm{eff}}\;\left(\theta \right)$ is calculated for stiffness-trend assessment. The complete measurement-to-property workflow and its implementation, including specimen fabrication, wavefield acquisition, directional frequency–wavenumber processing, dispersion-point extraction, regression formulation, and effective-modulus conversion, are described in Materials and Methods. These outputs provide direction-wise effective property estimates for the scanned region and form the basis for the subsequent assessment of directional stiffness variation.

### Benchmark verification using a 2024-T3 aluminum plate

A key risk in direction-resolved guided wave analysis is that apparent angular variation can arise from the measurement geometry, actuator directivity, finite scan window, edge reflections, or processing pipeline rather than from the material itself. To test this possibility, the complete workflow was first applied to a 2024-T3 aluminum plate, which provides a well-characterized isotropic benchmark with reference properties listed in Table S1. For this material, the expected signatures are circular wavefronts in the time–space domain, circular spectral rings in the frequency–wavenumber domain, and minimal angular variation in the recovered material properties.

The measured wavefields at 30, 40, 50, and 60 μs are shown in Fig. 2A to D. The wavefronts remain nearly circular as the wave packet expands, which is the expected real-space signature of isotropic in-plane guided wave propagation. A weaker and faster S_0_ component is visible ahead of the dominant A_0_ response in the early-time frames, whereas the A_0_ branch produces the stronger out-of-plane response. These observations confirm that the PZT-LDV system can excite and capture multimodal guided waves with sufficient spatial and temporal resolution for dispersion analysis.

**Fig. 2.**
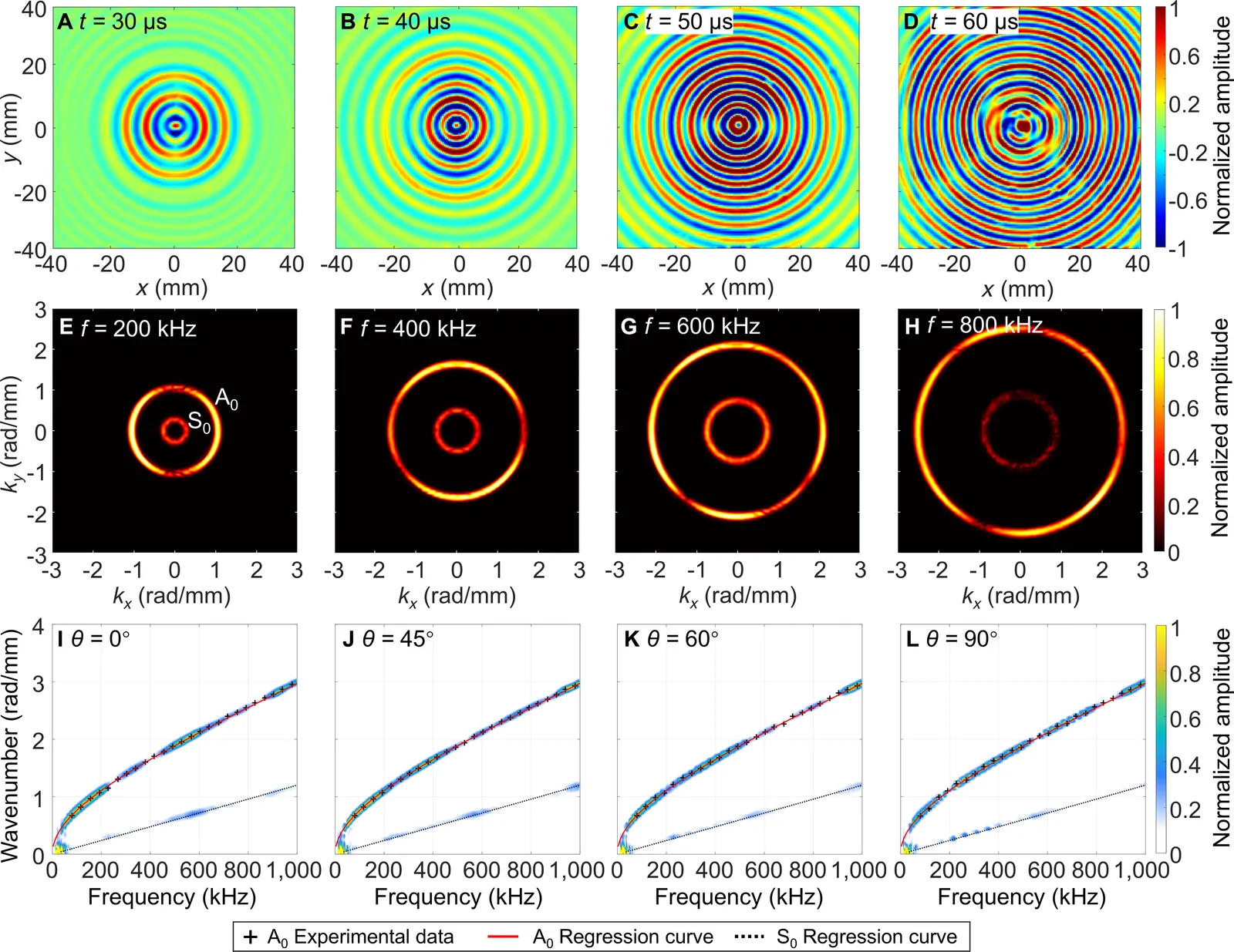
Benchmark verification using a 2024-T3 aluminum plate. (A to D) LDV-measured normalized out-of-plane velocity wavefields at 30, 40, 50, and 60 μs. (E to H) Normalized frequency–wavenumber spectra at 200, 400, 600, and 800 kHz. (I to L) Directional frequency–wavenumber profiles and dispersion curve regression results at θ = 0°, 45°, 60°, and 90°. Symbols denote extracted experimental dispersion points; curves denote fitted theoretical branches.

The same wavefield was transformed into the frequency–wavenumber domain using the directional analysis procedure described in Materials and Methods. Figure 2E to H shows the frequency–wavenumber spectra at 200, 400, 600, and 800 kHz. The spectral features form nearly concentric circular rings, confirming that for a fixed frequency, the measured wavenumber is approximately independent of in-plane propagation direction. Minor angular amplitude variations are attributed to finite scan-window effects, edge reflections, diffraction, scattering, and local mode interactions rather than intrinsic anisotropy.

Representative directional frequency–wavenumber profiles are shown in Fig. 2I to L. For each profile, local spectral maxima were extracted as experimental dispersion points and fitted using the regression procedure described in Materials and Methods. Because the measured out-of-plane response in the thin aluminum plate was dominated by the A_0_ branch, the aluminum benchmark regression used the A_0_ branch as the primary inversion input. The S_0_ feature is shown where visible but was not the primary constraint for this verification case.

The fitted dispersion curves agree closely with the extracted experimental points at the representative directions. Applying the same procedure to the principal 12-direction grid from 0° to 330° with 30° increments yielded an average shear-wave velocity of 3,159.12 ± 24.74 m/s and an average Poisson’s ratio of 0.33 ± 0.01. These values agree closely with the reference values of 3,173.63 m/s and 0.33 listed in Table S1. The limited angular scatter indicates that the workflow does not introduce substantial artificial anisotropy in an isotropic material. The aluminum benchmark is used here as a processing-chain control rather than as a universal material-stability test; it verifies that the measurement geometry and directional frequency–wavenumber processing do not create artificial angular anisotropy in a nominally isotropic plate.

### Guided wave measurements in the printed thermoset composite

After verifying unbiased isotropic recovery in the aluminum benchmark, the framework was applied to an additively manufactured chopped-carbon-fiber thermoset composite. The specimen was printed using a 0° zig-zag raster with 100% infill, and the positive x axis was defined along the print-path direction. The detailed fabrication procedure is described in Materials and Methods. This architecture is expected to produce direction-dependent guided wave behavior because wave propagation along the printed beads and across bead interfaces samples different effective mechanical pathways.

The experimental setup and scan-region definition are shown in Fig. 3A to C. Two front-surface scan windows were defined as ROI A and ROI B, and ROI A was additionally scanned from the back surface. These measurements produced 3 analyzed scan datasets: ROI A-front, ROI A-back, and ROI B-front. Because ROI A-back was acquired from the opposite surface, its local scan coordinates were registered to the same specimen-fixed coordinate system before directional slicing and cross-dataset averaging. This registration preserved the definition of *θ* relative to the print-path direction for all datasets.

**Fig. 3.**
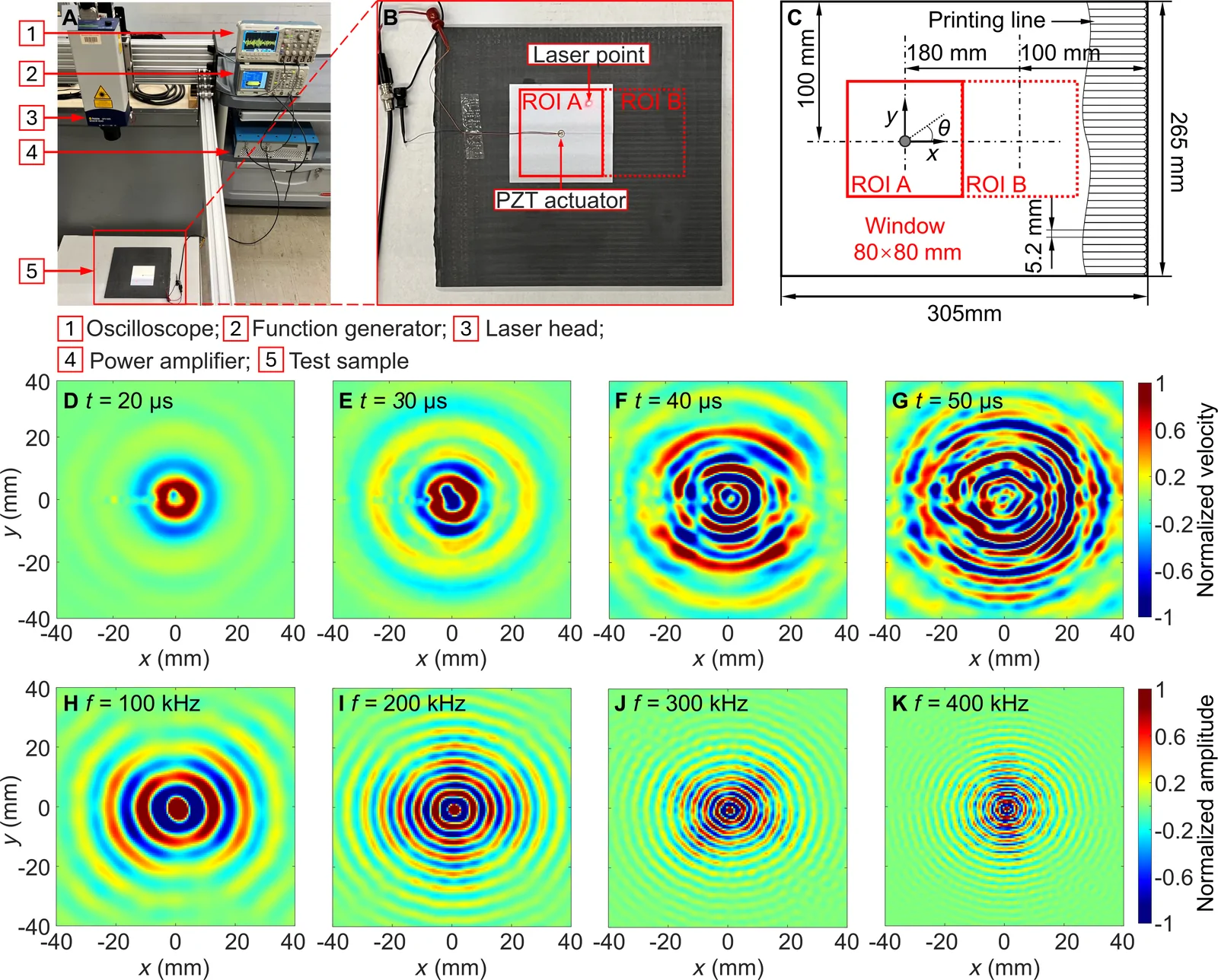
Composite scan-region definition and representative guided wavefields. (A) PZT-LDV measurement configuration for the printed composite specimen. (B) Printed thermoset composite specimen with the PZT actuator, laser point, and ROI A/ROI B scan windows. (C) Specimen geometry, coordinate system, print-line direction, and scan-window locations. (D to G) Representative LDV-measured normalized out-of-plane velocity wavefields from ROI A-front at 20, 30, 40, and 50 μs. (H to K) A_0_-dominant filtered wavefield components at 100, 200, 300, and 400 kHz.

Representative time–space wavefields from ROI A-front are shown in Fig. 3D to G. In contrast to the aluminum benchmark, the printed composite produces clearly elliptical rather than circular wavefronts. The wavefront extends farther along the *x* direction than along the *y* direction, indicating faster guided wave propagation along the print path than across the printed beads. This observation directly demonstrates that the printed composite cannot be represented by a single in-plane guided wave velocity or stiffness-related response.

The anisotropic wavefield is consistent with line-by-line printed architecture. Possible contributors include greater bead continuity along the print path, fiber-orientation bias induced by extrusion, inter-bead interfaces, local porosity, and transverse bonding variations. Because direct microstructural characterization was not performed in this study, these factors are interpreted as plausible contributors rather than as individually verified causes. The full-field wavefields in Fig. 3 provide direct evidence of direction-dependent guided wave propagation in the printed composite. Local defects or strong inhomogeneity would be embedded in the measured effective wave response and could perturb the fitted direction-wise properties through scattering, attenuation, or local velocity changes.

To further isolate the directional wave behavior, the measured response was transformed into the frequency–wavenumber domain and filtered around the high-amplitude A_0_ region, as described in Materials and Methods. The resulting A_0_-dominant filtered wavefield components are shown in Fig. 3H to K at 100, 200, 300, and 400 kHz. The elliptical propagation pattern remains evident across frequency, especially as the wavelength decreases at higher frequencies. Together, the time-domain and filtered wavefields demonstrate that the PZT-LDV system can acquire interpretable anisotropic guided wave responses in the printed thermoset composite.

### Directional frequency–wavenumber analysis and effective-property estimation

The elliptical wavefields in Fig. 3 qualitatively reveal print-path-dependent propagation, but mechanical-property characterization requires converting these wavefield features into quantitative directional dispersion and property estimates. The composite wavefield was therefore analyzed using the same frequency–wavenumber transformation and directional slicing procedure verified in the aluminum benchmark.

Figure 4A to D shows the frequency–wavenumber spectra of the printed composite at 100, 200, 300, and 400 kHz. Unlike the nearly circular spectral rings observed in the aluminum benchmark, the composite spectra exhibit elliptical high-amplitude features. For a given frequency, the wavenumber is smaller along the print-path direction and larger in the transverse direction. Because lower wavenumber at fixed frequency corresponds to higher phase velocity, the reciprocal-space spectra are consistent with the real-space wavefields in Fig. 3.

**Fig. 4.**
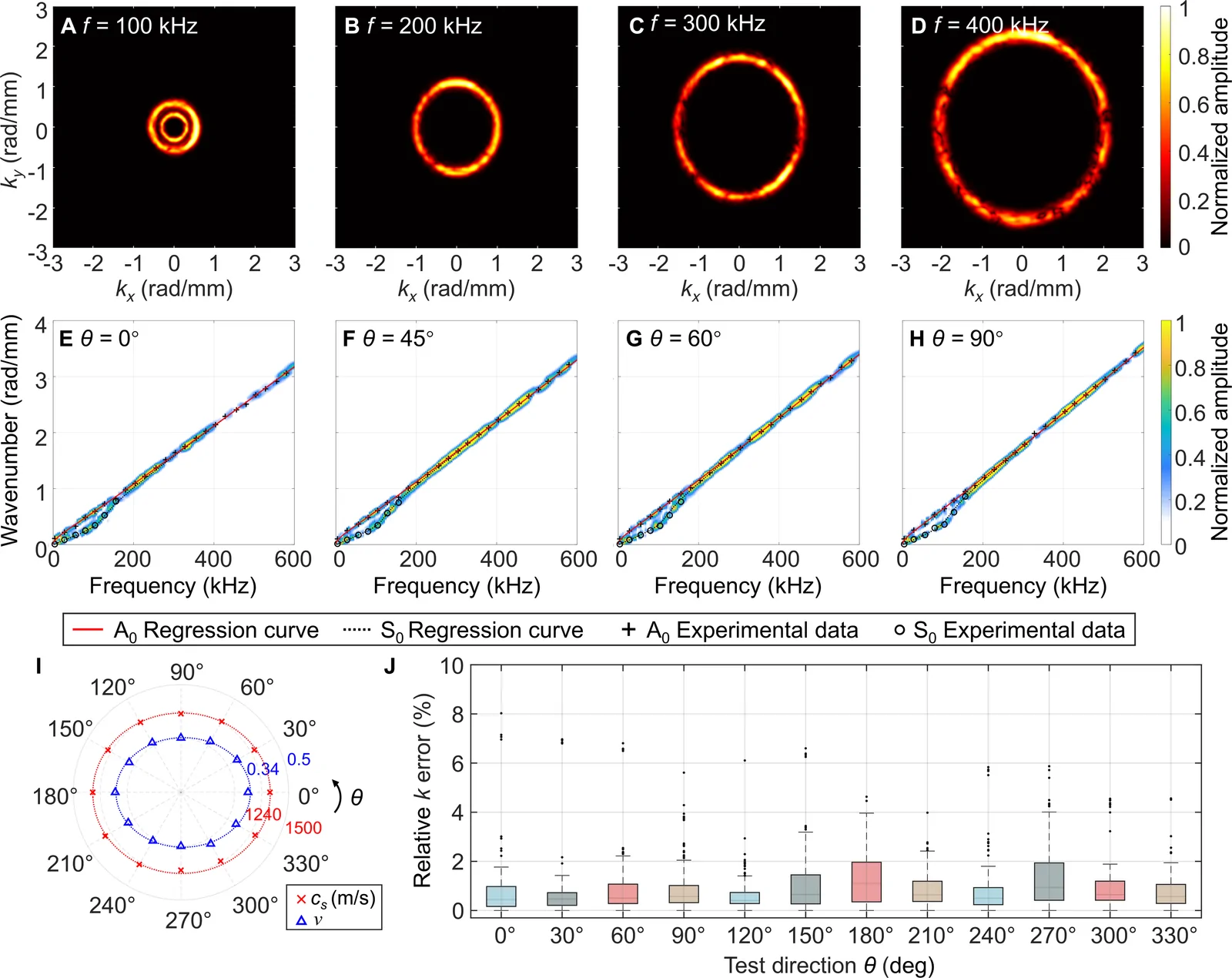
Directional frequency–wavenumber analysis and effective-property estimation for the printed thermoset composite. (A to D) Normalized frequency–wavenumber spectra at 100, 200, 300, and 400 kHz. (E to H) Directional frequency–wavenumber profiles and dispersion curve regression results at θ = 0°, 45°, 60°, and 90°. The 45° profile was additionally extracted for tensile comparison. Symbols denote extracted A_0_ and S_0_ dispersion points; curves denote fitted regression curves. (I) Direction-dependent c_s,eff_ (θ) and ν_eff_ (θ), averaged across ROI A-front, ROI A-back, and ROI B-front after coordinate registration. (J) Pooled relative wavenumber-error distributions by propagation direction.

Representative directional profiles extracted at 0°, 45°, 60°, and 90° are shown in Fig. 4E to H. The principal directional property map used the 12-direction grid from 0° to 330° with 30° increments. The 45° profile was additionally extracted only to support direct comparison with the ASTM D638 tensile result in the same direction. In the printed composite, the measured out-of-plane LDV response was dominated by the A_0_ branch. The S_0_ and A_0_ branches were spectrally separated at lower frequencies. Because the greater plate thickness placed the analyzed frequency range in a higher frequency-thickness regime, the 2 fundamental branches progressively approached the Rayleigh-wave asymptote as frequency increased, reducing their wavenumber separation [23]. Consequently, the S_0_ ridge became increasingly difficult to resolve from A_0_ at higher frequencies rather than physically disappearing as a propagating mode. Therefore, A_0_ was used as the primary inversion branch, whereas S_0_ points were retained only when they were clearly separated from A_0_ and satisfied the automated branch-continuity criterion described in Materials and Methods. The A_0_-only versus A_0_ + S_0_ sensitivity results in Tables S2 to S4 show that conditional S_0_ inclusion changed $c_{s,\mathrm{eff}}$ by at most 1.43%, $E_{\mathrm{eff}}$ by at most 2.39%, and $\nu_{\mathrm{eff}}$ by at most approximately 0.006 in absolute value, indicating that the primary directional trend was not controlled by the conditionally retained S_0_ points.

The extracted dispersion points align closely with the fitted theoretical curves across the representative directions, indicating that the direction-wise regression captures the measured frequency–wavenumber relationship in the printed composite. Applying this procedure to the principal 12-direction grid yielded direction-dependent $c_{s,\mathrm{eff}}$ and $\nu_{\mathrm{eff}}$ values for each scan dataset. The main directional distributions in Fig. 4I were obtained by averaging ROI A-front, ROI A-back, and ROI B-front after coordinate registration. Dataset-specific property maps and residual distributions are provided in Figs. S1 to S3, and the corresponding $c_{s,\mathrm{eff}}$, $\nu_{\mathrm{eff}}$, and $E_{\mathrm{eff}}$ statistics are provided in Tables S5 to S7. Using the same fitted $c_{s,\mathrm{eff}}$ and $\nu_{\mathrm{eff}}$, $E_{\mathrm{eff}}$ was calculated for each scan dataset and direction before cross-dataset averaging.

The recovered effective shear-wave velocity is highest near the print-path direction and lowest near the transverse direction. The mean $c_{s,\mathrm{eff}}$ is approximately 1,274 m/s at 0°, and 1,265 m/s at 180°, whereas it decreases to approximately 1,148 m/s at 90° and 1,140 m/s at 270°. The recovered effective Poisson’s ratio follows a similar trend, decreasing from approximately 0.349 at 0° to approximately 0.300 at 270°. These directional distributions provide a quantitative counterpart to the elliptical wavefronts and elliptical frequency–wavenumber spectra observed in Figs. 3 and 4A to D.

The residual-level stability of the dispersion regression is summarized in Fig. 4J. Relative wavenumber-error samples were pooled across ROI A-front, ROI A-back, and ROI B-front after coordinate registration. Dataset-specific residual distributions in Figs. S1 to S3 show similar behavior across the 3 scan datasets, supporting within-plate consistency of the recovered directional property trends. Across the 36 direction-dataset cases, the multistart convergence check yielded maximum near-best-solution spreads of 6.9 × 10^−6^% in $c_{s,\mathrm{eff}}$, 7.4 × 10^−7^ in absolute $\nu_{\mathrm{eff}}$, and 2.4 × 10^−5^% in $E_{\mathrm{eff}}$, demonstrating reproducible selection of the minimum-cost solution across initializations.

### Tensile comparison and angular stiffness-trend assessment

To evaluate whether the guided wave inversion captured mechanically meaningful directional stiffness contrast, the direction-wise estimates were compared with ASTM D638 tensile measurements. The mechanical tests were performed at 0°, 45°, 60°, and 90°, spanning the print-path, off-axis, and transverse directions. Five coupons were initially tested in each direction from the same batch of printed panels as the guided wave specimen. One 90° coupon exhibited premature failure within the grip region and was excluded, resulting in n=5 at 0°, 45°, and 60° and n=4 at 90°.

The dispersion curve regression returned $c_{s,\mathrm{eff}}\;\left(\theta \right)$ and $\nu_{\mathrm{eff}}\;\left(\theta \right)$, which were converted into the effective modulus estimate $E_{\mathrm{eff}}\;\left(\theta \right)$ using Eq. (15). This quantity follows the same direction-wise equivalent-isotropic parameterization used in the dispersion regression. Therefore, $E_{\mathrm{eff}}$ is interpreted as a guided wave-derived stiffness-related metric rather than as a full orthotropic Young’s modulus.

At the mechanically tested directions, the guided wave-derived E eff reproduced the tensile stiffness hierarchy, with both methods showing the highest stiffness near the print-path direction and lower stiffness toward the transverse direction. Specifically, the guided wave-derived $E_{\mathrm{eff}}$ decreased from approximately 4.60 GPa at 0° to 3.61 GPa at 90°, consistent with the tensile decrease from approximately 4.40 GPa to 3.81 GPa over the same angular range. The absolute discrepancies ranged from 2.16% to 5.41%. Because the guided wave datasets were obtained from one printed plate, whereas the tensile coupons were machined from 2 other panels produced in the same batch, and because the 2 methods probe different physical regimes, the comparison supports same-batch consistency in directional stiffness trend and approximate magnitude across the tested directions. The corresponding guided wave-derived and tensile results at the 4 mechanically tested directions are summarized in Table 1.

**Table 1.** Comparison of guided wave-derived effective stiffness metrics and ASTM D638 tensile moduli at the 4 mechanically tested directions. Values are reported as mean ± SD. The tensile-test sample sizes were *n* = 5 at 0°, 45°, and 60° and *n* = 4 at 90°; one 90° coupon was excluded because of premature failure within the grip region.

Test direction	0°	45°	60°	90°
*c*_*s*,eff_ (m/s)	1,273.803	1,210.109	1,186.139	1,148.339
*ν*_eff_	0.349	0.331	0.320	0.302
Guided wave-derived *E*_eff_ (GPa)	4.601 ± 0.333	4.093 ± 0.202	3.903 ± 0.245	3.607 ± 0.180
ASTM D638 *E* (GPa)	4.399 ± 0.161	4.233 ± 0.281	3.989 ± 0.223	3.813 ± 0.416
Discrepancies (%)	4.59%	3.31%	2.16%	5.41%

To assess whether the 12-direction guided wave-derived $E_{\mathrm{eff}}$ followed an angular stiffness trend consistent with the separately measured tensile data, the guided wave-derived $E_{\mathrm{eff}}$ values were compared with a tensile-constrained in-plane orthotropic stiffness surrogate constructed from the ASTM D638 mean moduli, as described in Materials and Methods. This surrogate was not fitted to the guided wave data and was used only as a tensile-derived angular stiffness reference.

As shown in Fig. 5A, the tensile-constrained reference captures the expected elliptical angular stiffness trend, with higher stiffness near the print-path direction and lower stiffness near the transverse direction. The 12-direction guided wave-derived $E_{\mathrm{eff}}$ values follow the same angular trend, and the ASTM D638 points fall near the corresponding reference curve at the mechanically tested directions. The shaded band represents the scan-dataset-level standard deviation of the guided wave-derived $E_{\mathrm{eff}}$, indicating the variability among ROI A-front, ROI A-back, and ROI B-front.

**Fig. 5.**
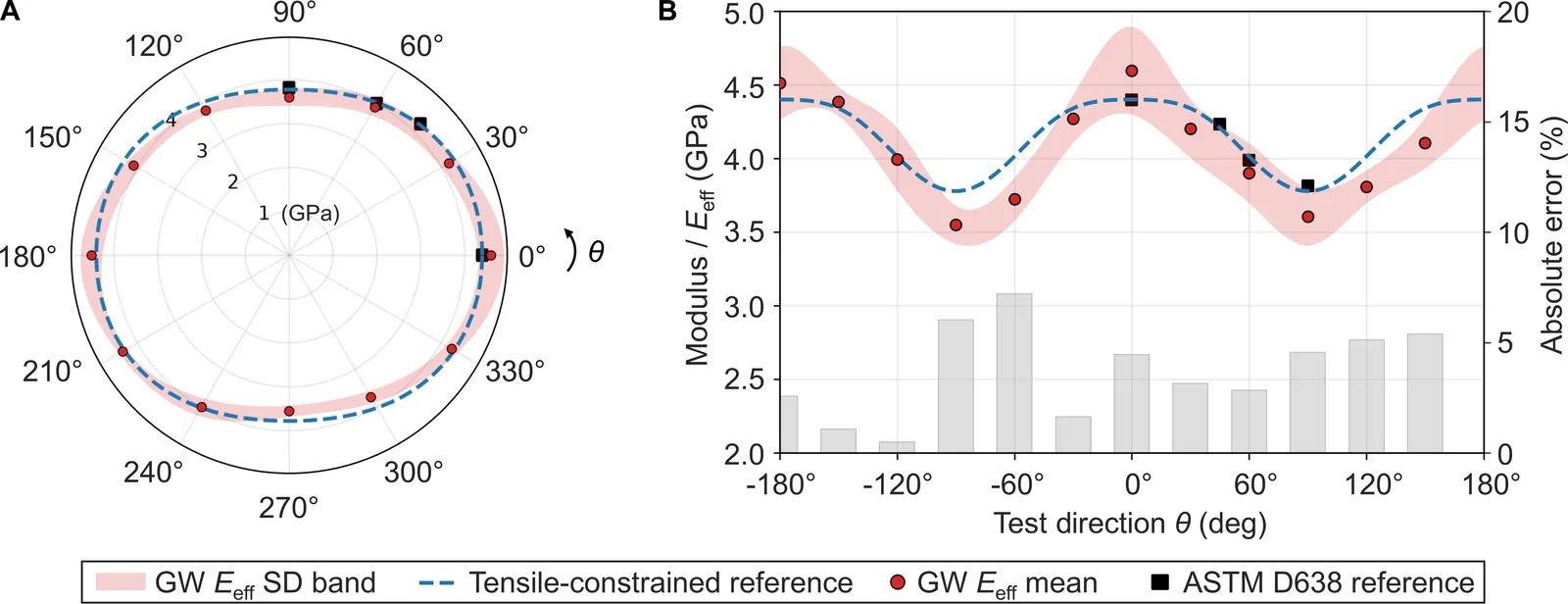
Trend-level comparison between the tensile-constrained orthotropic stiffness surrogate and 12-direction guided wave-derived *E*_eff_. (A) Polar comparison of the tensile-constrained reference, guided wave-derived *E*_eff_, and ASTM D638 tensile modulus. Red circles denote the mean guided wave-derived *E*_eff_, and the shaded band denotes ± SD from 3 scan datasets; black squares denote ASTM D638 results. (B) Wrapped angular comparison from −180° to 180°. Gray bars show the absolute percentage difference between guided wave-derived *E*_eff_ and the tensile-constrained reference.

Figure 5B presents the same comparison in a wrapped angular coordinate from −180° to 180° and includes the absolute percentage difference between the guided wave-derived $E_{\mathrm{eff}}$ and the tensile-constrained reference. The 12-direction guided wave estimates followed the tensile-constrained orthotropic trend with a root mean square error (RMSE) of 0.171 GPa, a mean absolute percentage difference of 3.71%, and a correlation coefficient of 0.94. The largest pointwise difference was 7.21% at 300°, while most directions remained within approximately 5% of the tensile-derived reference. Because the tensile-constrained reference was derived from 4 directional mean moduli, the 12-direction comparison serves as a trend-level assessment of angular stiffness consistency, with the RMSE, mean absolute percentage difference, and correlation coefficient quantifying agreement with the fitted reference.

## Discussion

This study establishes a PZT-LDV guided wave framework for direction-resolved effective mechanical-property characterization of additively manufactured thermoset composites. The central advance is the conversion of direction-resolved guided wave dispersion into directional effective stiffness estimates that are subsequently assessed against tensile measurements. By combining single-contact PZT excitation, noncontact scanning LDV wavefield acquisition, directional frequency–wavenumber analysis, and dispersion-curve regression, the framework links print-path-dependent guided wave behavior to effective shear-wave velocity $c_{s,\mathrm{eff}}\;\left(\theta \right)$, effective Poisson’s ratio $\nu_{\mathrm{eff}}\;\left(\theta \right)$, and guided wave-derived effective modulus $E_{\mathrm{eff}}\left(\theta \right)$.

The aluminum benchmark was an essential control because it tested whether the sensing and analysis workflow itself introduced artificial angular variation. This verification step is important because directional guided wave analysis can otherwise be confounded by actuator directivity, finite scan-window effects, boundary reflections, mode interactions, or processing artifacts. Together, the aluminum results indicate that the angular contrasts observed in the printed composite originate primarily from the specimen response rather than from the measurement geometry or directional processing workflow.

When applied to the printed thermoset composite, the framework revealed pronounced print-path-dependent guided wave behavior. The elliptical wavefields and frequency–wavenumber spectra are consistent with faster guided wave propagation along the print-path direction than across the printed beads. Possible contributors include greater bead continuity along the print path, extrusion-induced fiber-orientation bias, inter-bead interfaces, local porosity, and transverse bonding variations. Because direct microstructural characterization was not performed, these factors are interpreted as plausible contributors rather than individually verified causes. Directional dispersion-curve regression converted these signatures into direction-dependent effective property distributions. The consistency across ROI A-front, ROI A-back, and ROI B-front further supports the robustness of the direction-resolved characterization.

The guided wave-derived effective modulus estimates also reproduced the directional trend measured by ASTM D638 tensile testing. This agreement should be interpreted as practical agreement in directional stiffness trend and approximate magnitude, not as strict equivalence between dynamic guided wave-derived properties and quasi-static tensile moduli. The tensile-constrained in-plane orthotropic surrogate further shows that the 12-direction guided wave estimates preserve the dominant angular stiffness trend without implying recovery of a complete orthotropic stiffness tensor. Within this interpretation, the method provides a useful nondestructive estimate of direction-dependent stiffness-related behavior in the printed composite.

The proposed framework occupies a practical middle ground among destructive coupon testing, sparse guided wave sensing, and defect-oriented NDE. Destructive testing can provide direction-specific mechanical properties but is limited by coupon extraction, machining requirements, and low spatial coverage. Sparse guided wave sensing can monitor selected propagation paths but cannot resolve continuously varying directional behavior over a scanned region. Defect-oriented NDE, including CT, provides complementary information on discontinuities, porosity, and geometric fidelity but is not primarily intended to provide direction-resolved effective stiffness-response maps. The PZT-LDV framework provides this complementary route through a single bonded actuator for repeatable excitation and noncontact scanning LDV acquisition of dense wavefield data for direction-resolved stiffness-response estimation.

The model used here is reduced-order by design. Each directional dispersion profile is fitted independently using an equivalent-isotropic plate model. Within this formulation, $c_{s,\mathrm{eff}}\;\left(\theta \right)$ is the primary velocity-related descriptor, $E_{\mathrm{eff}}\left(\theta \right)$ is a derived stiffness-related metric for comparison with tensile trends, and $\nu_{\mathrm{eff}}\;\left(\theta \right)$ acts as an effective dispersion-shape parameter. Therefore, the recovered $c_{s,\mathrm{eff}}\;\left(\theta \right)$, $\nu_{\mathrm{eff}}\;\left(\theta \right)$, and $E_{\mathrm{eff}}\;\left(\theta \right)$ should be interpreted as effective guided wave properties associated with each propagation direction. They quantify directional mechanical contrast, but they do not constitute a full orthotropic stiffness tensor or a complete anisotropic constitutive reconstruction. This distinction is important: The current framework is designed for practical direction-resolved mechanical-property screening, not full anisotropic material identification. Tensor-level identification would require additional independent elastic constants and an anisotropic guided wave forward model.

The 3 scan datasets produced consistent directional patterns and residual behavior, while conditional $S_{0}$ inclusion had only a limited influence on the recovered parameters. Together with the multistart convergence results, these findings support the stability of the recovered directional trends across the analyzed datasets and the numerical consistency of regression, while the fitted parameters remain direction-specific effective descriptors rather than uniquely identified anisotropic material constants.

Several limitations remain. The present results demonstrate consistent directional responses across ROI A-front, ROI A-back, and ROI B-front within the tested plate; larger specimen sets and additional printing batches are needed to quantify specimen-to-specimen and batch-level variability. For full-scale LFAM tooling, the method can be deployed as a targeted regional inspection, with scan regions of specified dimensions placed at process-critical locations rather than covering the entire surface. Extension to curved, thick, thin-wall, lattice, or geometrically complex structures will require geometry-aware scan registration, local thickness information, and forward models matched to the local structural geometry. Because defects and strong inhomogeneity can alter scattering, attenuation, and local velocity, combining guided wave metrics with CT, microscopy, or other defect-characterization methods would help relate effective stiffness responses to underlying manufacturing features.

Future work should therefore focus on anisotropic dispersion-curve regression, larger specimen sets and printing batches, additional print architectures and process conditions, curved or nonplanar components, and in situ monitoring during manufacturing. For field deployment, scan-time reduction, line-of-sight planning, and selected critical-region or robotic scans should also be considered. Scanning LDV can be affected by surface roughness, optical reflectivity, and ambient vibration; these effects can be mitigated through reflective treatment, vibration control, signal averaging, and post-processing denoising. Thus, the results demonstrate that PZT-LDV full-field guided wave sensing, directional frequency–wavenumber analysis, and dispersion curve regression provide a practical nondestructive route for scan-region-level, direction-resolved property characterization to support quality control and structural evaluation workflows for LFAM thermoset composite tooling.

## Materials and Methods

### Study design

A benchmark-and-application design was used to evaluate the PZT-LDV guided wave framework for direction-resolved effective-property characterization of additively manufactured thermoset composites. The workflow was first applied to an isotropic 2024-T3 aluminum plate for method verification and then to a chopped-carbon-fiber thermoset composite for directional property estimation. Guided wave-derived effective stiffness estimates from the printed composite were compared with ASTM D638 tensile measurements in selected directions and with a tensile-constrained in-plane orthotropic stiffness surrogate for 12-direction angular trend assessment.

### Characterization workflow

As shown in Fig. 1A, the thermoset composite was fabricated by RAM, and the print-path direction was used as the angular reference for subsequent guided wave characterization. As illustrated in Fig. 1B, broadband guided waves generated by a bonded PZT actuator were measured over the prescribed scan region using scanning LDV to form the time–space wavefield $u\left(t,x,y\right)$. The measured wavefield was transformed into the frequency–wavenumber spectrum $U\left(f,k_{x},k_{y}\right)$, and directional slicing was used to obtain propagation-angle-specific profiles $V\left(\theta ,f,k\right)$, from which mode-labeled dispersion points were extracted. As shown in Fig. 1C, the dispersion data for each propagation direction were independently fitted using the equivalent-isotropic Rayleigh–Lamb formulation to obtain $c_{s,\mathrm{eff}}\;\left(\theta \right)$ and $\nu_{\mathrm{eff}}\;\left(\theta \right)$; the corresponding $E_{\mathrm{eff}}\;\left(\theta \right)$ was then calculated as a stiffness-related metric for mechanical comparison. The fabrication, sensing, directional processing, dispersion extraction, regression, and effective-modulus conversion procedures are described in the following subsections.

### PZT-LDV guided wave sensing system for time–space wavefield acquisition

The guided wave sensing system consisted of a piezoelectric actuator for guided wave excitation and a scanning laser Doppler vibrometer mounted on a 3-axis motorized motion stage for noncontact wavefield acquisition. A circular PZT actuator with a diameter of 7 mm and a thickness of 0.2 mm was bonded to the specimen surface using epoxy. Guided waves were excited by a broadband chirp signal generated by a function generator, amplified by a power amplifier, and supplied to the PZT actuator. The LDV laser beam was directed nearly normal to the specimen surface to measure the out-of-plane surface velocity response. The response was recorded by an oscilloscope and synchronized with the motion-control program through custom MATLAB scripts using the Instrument Control Toolbox [43].

For each laser position $\left(x_{i},y_{i}\right)$, the LDV recorded a waveform $u\left(t,x_{i},y_{i}\right)$. Point-by-point scanning over the prescribed area assembled the measured responses into the time–space wavefield $u\left(t,x,y\right)$. All scans used an 80 mm × 80 mm scan area, a 0.8-mm spatial step, a zig-zag scan path, and a 31.25-MHz acquisition rate. Ultrathin retro-reflective tape was applied to the scan area to improve laser return and signal-to-noise ratio. Temporal and spatial sampling parameters were selected to satisfy the Nyquist requirements for the frequency and wavenumber ranges analyzed in this study.

### Directional frequency–wavenumber analysis of measured time–space wavefields

Each wavefield $u\left(t,x,y\right)$ was transformed into the frequency–wavenumber domain using a multidimensional Fourier transform,

$$U\left(f,k_{x},k_{y}\right)=∭u\left(t,x,y\right)e^{-i\left(2\pi \mathrm{ft}-k_{x}x-k_{y}y\right)}\;dtdxdy$$ (1)

where *f* is the wave frequency; $k_{x}$ and $k_{y}$ are the wavenumbers along the *x* and *y* directions, respectively; and $U\left(f,k_{x},k_{y}\right)$ is the resulting frequency–wavenumber spectrum. To characterize guided wave behavior as a function of propagation direction, directional slicing was performed on the full frequency–wavenumber spectrum. The in-plane wavenumber components were related to the overall wavenumber *k* and propagation angle *θ* by

$$k_{x}=k\cos\theta ,k_{y}=k\sin\theta ,k=\sqrt{{k_{x}}^{2}+{k_{y}}^{2}}.$$ (2)

Using these relationships, directional profiles $V\left(\theta ,f,k\right)$ were extracted from $U\left(f,k_{x},k_{y}\right)$. The primary directional analysis was performed at 12 propagation directions from 0° to 330° with 30° increments. An additional 45° direction was extracted only for tensile-comparison purposes.

For each directional profile, experimental dispersion points were extracted automatically on a 2-kHz frequency grid. For the printed thermoset composite, theoretical A_0_ and S_0_ reference branches were calculated from the Rayleigh–Lamb equations [Eqs. (3) to (5)], using the reported plate thickness of 7.9 mm and the composite initial parameters in Table S8 ($c_{s}$ = 1,250 m/s and ν = 0.30). These reference branches were used only to initialize ridge tracking. For each mode, frequencies were examined in ascending order, and the initial ridge point was selected as the highest-amplitude local spectral maximum satisfying the mode-specific acceptance criteria below within ±0.25 rad/mm of the corresponding reference branch, at the first frequency where such a maximum was available.

For the printed thermoset composite, A_0_ candidates were evaluated at 300 positive-frequency samples over the analyzed 2- to 600-kHz range by selecting the highest-amplitude eligible local maximum of the normalized directional spectrum $|V\left(\theta ,f,k\right)|$ within a ±0.25 rad/mm local search window around the most recently accepted ridge point, subject to finite k>0 and normalized confidence ≥ 0.1. The normalized confidence was defined as $C_{i}=|V\left(\theta ,f_{i},k_{i}\right)|/\max_{k}|V\left(\theta ,f_{i},k\right)|.$ After initialization, branch continuity was enforced by requiring $\left|k_{i}-k_{p}\right|\leq 0.25$ rad/mm, where $k_{p}$ denotes the most recently accepted wavenumber. A frequency sample was skipped if no eligible local maximum was found, and the search-window center was updated only after a point was accepted. This branch-guided ridge extraction reduced the selection of side-lobe, reflection-related, or mode-overlap features.

In the printed thermoset composite, A_0_ also remained the primary inversion branch, whereas S_0_ candidates were identified using the same automated branch-guided ridge-extraction procedure and retained only when the normalized confidence was ≥ 0.1, the candidate satisfied the same continuity criterion after initialization, and its separation from the A_0_ point was at least 0.1 rad/mm at the same sampled frequency. The A_0_–S_0_ separation criterion was also applied when selecting the initial S_0_ point. No post hoc manual deletion or re-selection of dispersion points was applied in the final auditable dataset. Ridge initialization and all subsequent point acceptance or rejection were performed automatically. The resulting experimental dataset for each direction was a set of mode-labeled dispersion points $\left\{\left(f_{i},k_{\exp,i},m_{i}\right)\right\}$, where $m_{i}\in \left\{A_{0},S_{0}\right\}$.

To quantify the influence of conditional S_0_ inclusion, the composite regressions were repeated using A_0_-only input and compared with the A_0_ + S_0_ regressions used for the main results. The resulting sensitivity values are reported in Tables S2 to S4.

For visualization of the composite wavefield, an additional wavenumber-domain filter was applied around the high-amplitude A_0_ region of the composite spectrum to isolate A_0_-dominant wavefield components.

### Dispersion curve regression for mechanical-property characterization

For each propagation direction, the experimentally extracted dispersion data were fitted independently using an equivalent-isotropic plate model. The resulting shear-wave velocity and Poisson’s ratio are therefore interpreted as direction-wise effective guided wave properties rather than as a complete anisotropic constitutive description. Similar direction-wise treatments have been reported in guided wave studies of fully filled printed plates [44].

Under the isotropic plate assumption, the guided wave dispersion behavior is governed by the Rayleigh–Lamb equations. For symmetric modes,

$$\frac{\tan\left(\mathrm{qh}\right)}{\tan\left(\mathrm{ph}\right)}=-\frac{4k^{2}\mathrm{pq}}{{\left(q^{2}-k^{2}\right)}^{2}}$$ (3)

and for antisymmetric modes,

$$\frac{\tan\left(\mathrm{qh}\right)}{\tan\left(\mathrm{ph}\right)}=-\frac{{\left(q^{2}-k^{2}\right)}^{2}}{4k^{2}\mathrm{pq}}$$ (4)

where

$$p^{2}=\frac{\omega^{2}}{c_{L}^{2}}-k^{2},q^{2}=\frac{\omega^{2}}{c_{s}^{2}}-k^{2},\omega =2\pi f$$

Here, *h* is half the plate thickness, *c*_L_ is the longitudinal-wave velocity, *c*_s_ is the shear-wave velocity, and *ν* is Poisson’s ratio. For isotropic materials,

$$c_{L}=c_{s}\sqrt{\frac{2\left(1-\nu \right)}{1-2\nu }}.$$ (5)

Accordingly, when the plate thickness is known, the theoretical dispersion curves can be parameterized by *c*_s_ and *ν*, which provides the physical basis for mechanical-property inversion from experimentally measured guided wave dispersion data. The sensitivity of the theoretical dispersion curves to $c_{s}$ and ν is illustrated in Fig. S4. In this parameterization, $c_{s}$ and ν were used as the independent regression variables because $c_{L}$ is constrained by the isotropic relation in Eq. (5) rather than fitted independently. The effective modulus $E_{\mathrm{eff}}$ was therefore treated as a derived stiffness-related quantity for tensile comparison, not as an independent regression parameter.

To fit the experimental dispersion data, a nonlinear least-squares dispersion curve regression solved with the Levenberg–Marquardt (LM) algorithm was performed [45]. The overall regression workflow is summarized in Fig. S5. For a given propagation direction and scan dataset, let the mode-labeled experimental dispersion set be written as

$$D={\left\{\left(f_{i},k_{\exp,i},m_{i}\right)\right\}}_{i=1}^{N}$$ (6)

where *m_i_ ∈* {A_0_, S_0_} denotes the mode label of the *i*th experimental point. The parameter vector was defined as *W* = [*c*_s_*, ν*]. For each experimental point, the corresponding theoretical wavenumber was computed as

$$k_{\text{theo},i}\left(W\right)=k_{\text{theo}}\left(f_{i};W,m_{i}\right)$$ (7)

by numerically solving the Rayleigh–Lamb equations [Eqs. (3) and (4)] for the corresponding A_0_ or S_0_ branch at frequency *f_i_*. Root tracking was performed branch-wise from low to high frequency so that the same physical branch was followed continuously during each optimization run.

For the printed thermoset composite, residuals from the available A_0_ points and the resolved S_0_ points were stacked directly into the same residual vector without additional branch-specific weighting. Consequently, the S_0_ branch acted as a supplementary constraint primarily through its conditional inclusion and smaller point count. The residual vector was therefore defined as

$$r\left(W\right)={\left[r_{1}\left(W\right),r_{2}\left(W\right),\ldots ,r_{N}\left(W\right)\right]}^{T},$$ (8)

with

$$r_{i}\left(W\right)=k_{\exp,i}-k_{\text{theo},i}\left(W\right),\;i=1,\ldots ,N$$ (9)

The regression objective was to minimize the sum of squared residuals,

$$L\left(W\right)={\left\|r\left(W\right)\right\|}_{2}^{2}=\sum_{i=1}^{N}r_{i}^{2}\left(W\right)$$ (10)

At iteration *t*, the LM increment Δ*W_t_* was obtained by solving

$$\left(J_{t}^{T}J_{t}+\lambda_{t}I\right)\Delta W_{t}=-J_{t}^{T}r\left(W_{t}\right)$$ (11)

where *J_t_ = ∂r/∂W* is the Jacobian matrix and *λ_t_* > 0 is the damping parameter. The parameter vector was then updated as

$$W_{\left(t+1\right)}=W_{t}+\Delta W_{t}$$ (12)

Upon convergence, the fitted wavenumber corresponding to the *i*th experimental point was defined as

$$k_{\mathrm{fit},i}=k_{\text{theo}}\left(f_{i};W_{\mathrm{opt}},m_{i}\right),$$ (13)

where *W*_opt_ denotes the optimized parameter vector. In the present implementation, the Jacobian was evaluated numerically, and the regression was solved in MATLAB (R2024b, MathWorks) using lsqnonlin with the LM algorithm. Separate initial values and admissible bounds were used for the aluminum benchmark and the printed-composite regressions. These settings are reported in Table S8. In the aluminum benchmark, the initial search was centered on the known isotropic reference values. In the printed composite, the regression was performed independently for each direction and each scan dataset using composite-specific initialization and bounds chosen to encompass the experimentally relevant property range. A multistart convergence check was performed for each scan dataset at the 12 principal propagation directions from $0^{\circ }$to $330^{\circ }$ with $30^{\circ }$ increments. Each direction-dataset case was evaluated using 25 initial parameter pairs arranged on a 5 × 5 evenly spaced grid over $c_{s}\in$ [1,050, 1,450] m/s and ν∈[0.22, 0.38], resulting in 36 direction-dataset cases and 900 regressions in total. For each direction-dataset case, $L_{\min}$ was defined as the lowest finite cost among runs classified as converged by the solver. Solutions satisfying $L\leq 1.001L_{\min}$ were classified as near-best solutions, and the minimum-cost solution was retained for reporting. Each near-best solution set contained solutions recovered from multiple initializations. Relative spreads in $c_{s,\mathrm{eff}}$ and $E_{\mathrm{eff}}$ were calculated as the range of the near-best solutions normalized by the corresponding minimum-cost estimate, whereas the absolute range was used for $\nu_{\mathrm{eff}}$.

### 2024-T3 aluminum benchmark specimen and test conditions

A 2024-T3 aluminum plate (Aerospace Metals LLC, USA) was used as the reference specimen for framework verification. The plate measured 200 mm × 180 mm with a thickness of 0.75 mm. Manufacturer-reported density, elastic modulus, shear modulus, and Poisson’s ratio, together with the corresponding calculated shear-wave velocity, are listed in Table S1 [46].

For the aluminum verification experiment, the *x* axis was defined along the long edge of the plate, and *θ* was defined as the angle between the guided wave propagation direction and the *x* axis. The scan area was 80 mm × 80 mm and was centered near the actuator. Guided waves were excited using a broadband 0- to 1-MHz chirp burst with a duration of 100 μs. A 15% Hann window was applied at both ends of the chirp to reduce spectral leakage. The excitation signal was amplified to approximately 20 Vpp. The aluminum experimental setup and specimen geometry are shown in Fig. S6, and the aluminum chirp signal and excitation spectrum are shown in Fig. S7A and B.

### Thermoset composite additive manufacturing and scan-dataset definition

Thermoset composite specimens were manufactured using a commercial MVP RAM-48 RAM system. The printing process consisted of 3 main stages. First, resin premixed with chopped carbon fibers and a separate catalyst/initiator stream were metered from individual reservoirs. Intermittent recirculation and vacuum degassing were used to maintain fiber suspension and stable feed delivery during printing. Second, the 2 streams were statically mixed approximately 25 mm upstream of a 5-mm nozzle and deposited layer by layer by a 3-axis gantry. Third, the printed panels were post-cured at 180 °C for at least 2 h, then face-milled and surface-ground to the final thickness.

Three square panels with nominal in-plane dimensions of 355.6 mm × 355.6 mm and nominal thickness of 12 mm were printed from PRD1647 (Polynt Composites, USA) containing 20 wt % short carbon fibers. All panels were printed using a 0° zig-zag raster with 100% infill. After post-processing, the final thickness was approximately 7.9 mm. One panel was used for guided wave measurements, whereas the remaining 2 panels were machined into ASTM D638 coupons for tensile validation. The density of the printed composite, determined by the Archimedes principle, was approximately 1,050 kg/m^3^.

For guided wave characterization, the printed specimen was cut to 305 mm × 265 mm to fit the LDV scanning platform. The plate thickness was measured at 5 locations using a micrometer (resolution 0.0001 inch), yielding a mean thickness of 7.883 ± 0.038 mm. The numerical thickness used in all composite Rayleigh–Lamb regressions was 7.9 mm. The composite density was measured by hydrostatic weighing based on the Archimedes principle. The air mass was 668.7 g, and 3 independent immersion measurements gave an apparent submerged mass of 31.6 ± 0.8 g, corresponding to a density of 1,049.6 ± 1.3 kg/m^3^; a density of 1,050 kg/m^3^ was used in the $E_{\mathrm{eff}}$ calculation. The positive *x* axis was defined along the print-path direction, and *θ* was defined as the angle between the guided wave propagation direction and the positive *x* axis. Two front-surface scan windows were defined as ROI A and ROI B, and ROI A was additionally scanned from the back surface. These datasets were denoted ROI A-front, ROI A-back, and ROI B-front. ROI A-front and ROI A-back represent measurements of the same physical region from opposite surfaces, whereas ROI B-front represents a second physical region on the same printed plate. These 3 datasets were therefore used to evaluate within-plate consistency across measurement surface and scan region. Their physical locations are defined in Fig. 3.

Because ROI A-back was measured from the opposite surface, its local measurement coordinates were transformed into the specimen-fixed global coordinate system before directional slicing, regression, and cross-dataset averaging. The coordinate registration was

$$x_{g}=-x_{\text{back}},\;y_{g}=y_{\text{back}}$$ (14)

This registration allowed ROI A-front, ROI A-back, and ROI B-front to share the same definition of θ. Dataset-specific property maps and residual distributions are provided in Figs. S1 to S3, and the corresponding $c_{s,\mathrm{eff}}$, $\nu_{\mathrm{eff}}$, and $E_{\mathrm{eff}}$ parameter statistics are provided in Tables S5 to S7.

Composite guided wave tests used the same PZT-LDV sensing and scanning procedure described above. Because the printed composite plate was thicker than the aluminum benchmark and attenuated high-frequency guided wave components more strongly, a broadband 0- to 800-kHz chirp burst with a duration of 100 μs was used. A 15% Hann window was applied at both ends of the chirp, and the drive voltage was increased to 40 Vpp. The composite excitation waveform and normalized spectrum are shown in Fig. S7C and D.

### Guided wave effective-modulus conversion and regression-error quantification

The dispersion curve regression returned $c_{s,\mathrm{eff}}\left(\theta \right)$ and $\nu_{\mathrm{eff}}\left(\theta \right)$ for each scan dataset and propagation direction. To obtain a stiffness-related quantity for comparison with tensile measurements, the fitted guided wave parameters were converted into a direction-wise equivalent-isotropic effective modulus as

$$E_{\mathrm{eff}}\left(\theta \right)=2\rho c_{s,\mathrm{eff}}^{2}\left(\theta \right)\left[1+\nu_{\mathrm{eff}}\left(\theta \right)\right],$$ (15)

where *ρ* is the measured density of the printed composite. This expression follows the same direction-wise equivalent-isotropic assumption used in the dispersion regression. Therefore, $E_{\mathrm{eff}}\left(\theta \right)$ is interpreted as a guided wave-derived, direction-dependent effective stiffness estimate, rather than as a fully reconstructed orthotropic Young’s modulus.

For the printed composite, $E_{\mathrm{eff}}$ was calculated for each scan dataset and direction using the corresponding fitted $c_{s,\mathrm{eff}}$ and $\nu_{\mathrm{eff}}$. Directional mean values and standard deviations of $c_{s,\mathrm{eff}}$, $\nu_{\mathrm{eff}}$, and $E_{\mathrm{eff}}$ were then obtained from ROI A-front, ROI A-back, and ROI B-front after coordinate registration.

The stability of the regression was quantified by calculating the relative wavenumber error for each experimentally extracted, mode-labeled dispersion point:

$$\varepsilon_{k}=\frac{|k_{\exp,i}-k_{\mathrm{fit},i}|}{k_{\exp,i}}\times 100\%,$$ (16)

where $k_{\exp,i}$ is the experimentally extracted wavenumber and $k_{\mathrm{fit},i}$ is the fitted theoretical wavenumber at the same frequency and mode label, as defined in Eq. (13). Error samples from ROI A-front, ROI A-back, and ROI B-front were pooled by propagation direction after coordinate registration to construct the main-text regression-error summary. Dataset-specific error distributions are reported in Figs. S1 to S3.

### Tensile validation and statistical analysis

External validation was performed using ASTM D638 tensile testing at 0°, 45°, 60°, and 90°[47]. Five coupons were tested in each direction using a universal testing machine (Shimadzu, Japan). The coupons were taken from the same batch of printed thermoset composite panels as the guided wave specimen to minimize fabrication-induced variability between the nondestructive and destructive test groups. Each coupon was machined to a nominal thickness of 7.0 ± 0.1 mm using a surface/thickness planer, with a narrow-section width of 13 mm and a gauge length of 50 mm. Tensile tests were conducted at a crosshead speed of 5 mm/min, and strain was measured using a biaxial clip-on extensometer (Instron, USA); the axial strain channel was used for tensile-modulus calculation.

The tensile modulus was reported as the mean ± standard deviation from 5 valid coupons at 0°, 45°, and 60° and 4 valid coupons at 90°; one 90° coupon was excluded because it exhibited premature failure within the grip region rather than the gauge section. The guided wave-derived effective modulus was compared with the corresponding tensile modulus using the absolute percentage discrepancy,

$$\text{Discrepancy}=\frac{|{\overset{-}{E}}_{\mathrm{eff}}-E_{\text{tensile}}|}{E_{\text{tensile}}}\times 100\%$$ (17)

where ${\overset{-}{E}}_{\mathrm{eff}}$ is the mean guided wave-derived effective modulus calculated using Eq. (15), and *E*_tensile_ is the mean ASTM D638 tensile modulus in the same direction.

### Tensile-constrained orthotropic stiffness-trend surrogate

To provide an anisotropic stiffness-trend reference for the 12-direction guided wave-derived $E_{\mathrm{eff}}$ values, a tensile-constrained in-plane orthotropic surrogate was constructed under a plane-stress assumption. For this surrogate, the print-path direction (0°) and transverse direction (90°) were taken as the principal orthotropic axes 1 and 2, respectively. In these principal axes, the normal-shear coupling terms $S_{16}$ and $S_{26}$ vanish, and the reduced plane-stress compliance relation can be written as [48]

$$\left[\begin{array}{c} \varepsilon_{1}\\ \varepsilon_{2}\\ \gamma_{12} \end{array}\right]=\left[\begin{array}{ccc} S_{11} & S_{12} & 0\\ S_{12} & S_{22} & 0\\ 0 & 0 & S_{66} \end{array}\right]\left[\begin{array}{c} \sigma_{1}\\ \sigma_{2}\\ \tau_{12} \end{array}\right]$$ (18)

where $S_{11}=1/E_{1}$, $S_{22}=1/E_{2}$, $S_{12}=-\nu_{12}/E_{1}=-\nu_{21}/E_{2}$, and $S_{66}=1/G_{12}$. For uniaxial loading at an angle θ relative to the principal 1-axis, transformation of the in-plane compliance relation gives the apparent directional modulus

$$\frac{1}{E\left(\theta \right)}=S_{11}\cos^{4}\theta +S_{22}\sin^{4}\theta +\left(2S_{12}+S_{66}\right)\sin^{2}\theta \cos^{2}\theta .$$ (19)

The 3 surrogate parameters were determined by weighted nonlinear least-squares fitting to the 4 ASTM D638 mean moduli, with each modulus residual normalized by the corresponding tensile-test standard deviation, equivalent to inverse-variance weighting. The tensile-constrained fit to the ASTM D638 mean moduli at 0°, 45°, 60°, and 90° yielded *E*_1_ = 4.40 GPa, *E*_2_ = 3.78 GPa, and 2*S*_12_ + *S*_66_ = 0.459 GPa^−1^. Because directional tensile modulus data alone identify only the combined term 2*S*_12_ + *S*_66_, *S*_12_ and *S*_66_ cannot be uniquely separated. Therefore, this surrogate was used only as a tensile-derived angular stiffness reference. It was not fitted to the guided wave data and was not intended to identify a complete orthotropic stiffness tensor.

## Data Availability

Relevant data supporting the key findings of this study are available within the article and the Supplementary Materials file. All raw data generated during the current study are available from the corresponding author upon request.
