# Supplementary material for: Laser Vibrometry and Dispersion Analysis-Enabled Mechanical Property Characterization for Additively Manufactured Thermoset Composites
Source: Research (Wash D C). 2026 Oct 19;9:1455. doi: 10.34133/research.1455 (PMC13678087; doi:10.34133/research.1455)
Supplement: Supplementary file 1 [file research.1455.f1.docx]

# SUPPLEMENTARY MATERIALS

## Supplementary Figures

**Fig. S1. Dataset-specific directional property and regression-error results for ROI A-front. (A)** Direction-dependent effective shear-wave velocity $c_{s,eff} (\theta)$ and Poisson’s ratio $\nu_{eff} (\theta)$ identified from ROI A-front. **(B)** Relative wavenumber-error distributions by test direction for ROI A-front.

**Fig. S2. Dataset-specific directional property and regression-error results for ROI A-back. (A)** Direction-dependent effective shear-wave velocity $c_{s,eff}(\theta)$ and Poisson’s ratio$\nu_{eff} (\theta)$ identified from ROI A-back after coordinate registration to the specimen-fixed global coordinate system. **(B)** Relative wavenumber-error distributions by test direction for ROI A-back.

**Fig. S3. Dataset-specific directional property and regression-error results for ROI B-front. (A)** Direction-dependent effective shear-wave velocity $c_{s,eff}(\theta)$ and Poisson’s ratio $\nu_{eff} (\theta)$ identified from ROI B-front. **(B)** Relative wavenumber-error distributions by test direction for ROI B-front.

**Fig. S4. Sensitivity of theoretical guided-wave dispersion curves to** $\boldsymbol{c}_{\boldsymbol{s}}$ **and** $\boldsymbol{\nu}$**. (A)** Influence of shear-wave velocity on the theoretical guided-wave dispersion curves with Poisson’s ratio fixed at $\nu$=0.3. **(B)** Influence of Poisson’s ratio on the theoretical guided-wave dispersion curves with shear-wave velocity fixed at c_s_=1200 m/s. The results show that the high-frequency portions of the $A_{0}$ and $S_{0}$ branches are more sensitive to variations in $c_{s}$, and the low-frequency portion of the $S_{0}$ branch is more sensitive to $\nu$.

**Fig. S5.** **Levenberg-Marquardt-based dispersion-curve regression workflow used for directional mechanical-property characterization.** Experimental dispersion points extracted from the directional frequency-wavenumber profiles are imported as the regression input. Initial material parameters and admissible bounds are specified, theoretical dispersion curves are generated, and the residual vector is evaluated from the mismatch between experimental and theoretical wavenumbers. The parameter vector $W=[c_{s},\nu]$ is then updated iteratively using the Levenberg-Marquardt algorithm until convergence is reached or the maximum iteration count is met.

**Fig. S6.** **PZT-LDV experimental setup for aluminum benchmark testing. (A)** Photograph of the PZT-LDV guided-wave measurement system used for the aluminum benchmark, including the oscilloscope, function generator, laser head, power amplifier, and test sample. **(B)** Aluminum benchmark plate geometry showing the PZT actuator, laser measurement point, 80 mm × 80 mm scan area, specimen dimensions, and coordinate definition used for directional frequency-wavenumber analysis.

**Fig. S7.** **Excitation signals used for the PZT-LDV guided-wave experiments. (A)** Time-domain voltage waveform of the broadband chirp excitation used for the 2024-T3 aluminum benchmark. **(B)** Corresponding normalized amplitude spectrum of the aluminum excitation signal. **(C)** Time-domain voltage waveform of the broadband chirp excitation used for the additively manufactured thermoset composite experiment. **(D)** Corresponding normalized amplitude spectrum of the composite excitation signal. The aluminum benchmark used a 0-1 MHz chirp, whereas the thicker and more attenuative composite specimen was excited using a 0-800 kHz chirp to emphasize the frequency range with reliable guided-wave response.

## Supplementary Tables

**Table S1.** Reference properties of the 2024-T3 aluminum reference plate used for benchmark verification. Density, elastic modulus, shear modulus, and Poisson’s ratio were taken from a 2024-T3 aluminum datasheet [main-text Ref. 46]. The plate thickness was measured from the benchmark specimen. The shear-wave velocity was calculated as $c_{s}=\sqrt{G/\rho}$.

| **Density (kg/m³)** | **Thickness (mm)** | **Elastic modulus (GPa)** | **Shear modulus (GPa)** | **Poisson's ratio** | **Shear wave velocity (m/s)** |
| --- | --- | --- | --- | --- | --- |
| 2780 | 0.75 | 73.1 | 28.0 | 0.33 | 3173.63 |

**Table S2.** S_0_-mode inclusion sensitivity for ROI A-front.

| **Direction** | **N (A_0_/S_0_)** | ***c_s_*_,eff_**  **A_0_-only / A_0_+S_0_  (m/s)** | **Δ *c_s_*_,eff_  (%)** | ***ν*_eff_**  **A_0_-only / A_0_+S_0_** | **Δ *ν*_eff_**  **(%)** | ***E*_eff_ A_0_-only / A_0_+S_0_ (GPa)** | **Δ *E*_eff_  (%)** |
| --- | --- | --- | --- | --- | --- | --- | --- |
| 0° | 300/89 | 1321.223 / 1320.934 | -0.022 | 0.358 / 0.360 | 0.669 | 4.977 / 4.983 | 0.133 |
| 30° | 300/83 | 1249.911 / 1244.907 | -0.400 | 0.351 / 0.353 | 0.614 | 4.432 / 4.403 | -0.641 |
| 60° | 300/80 | 1219.803 / 1219.351 | -0.037 | 0.334 / 0.335 | 0.158 | 4.170 / 4.168 | -0.035 |
| 90° | 300/77 | 1142.149 / 1154.080 | 1.045 | 0.317 / 0.311 | -1.774 | 3.607 / 3.667 | 1.665 |
| 120° | 300/79 | 1185.664 / 1194.099 | 0.711 | 0.322 / 0.319 | -1.037 | 3.904 / 3.950 | 1.171 |
| 150° | 300/80 | 1193.360 / 1206.443 | 1.096 | 0.341 / 0.336 | -1.569 | 4.011 / 4.084 | 1.797 |
| 180° | 300/86 | 1310.251 / 1303.776 | -0.494 | 0.355 / 0.357 | 0.634 | 4.884 / 4.844 | -0.821 |
| 210° | 300/84 | 1277.181 / 1270.198 | -0.547 | 0.330 / 0.333 | 0.876 | 4.556 / 4.516 | -0.876 |
| 240° | 300/83 | 1224.849 / 1234.766 | 0.810 | 0.324 / 0.320 | -1.193 | 4.171 / 4.226 | 1.329 |
| 270° | 300/74 | 1124.281 / 1124.371 | 0.008 | 0.310 / 0.310 | 0.000 | 3.477 / 3.478 | 0.016 |
| 300° | 300/77 | 1153.298 / 1166.836 | 1.174 | 0.335 / 0.329 | -1.685 | 3.728 / 3.800 | 1.929 |
| 330° | 300/84 | 1260.924 / 1259.461 | -0.116 | 0.350 / 0.351 | 0.272 | 4.508 / 4.500 | -0.162 |

**Table S3.** S_0_-mode inclusion sensitivity for ROI A-back.

| **Direction** | **N (A_0_/S_0_)** | ***c_s_*_,eff_**  **A_0_-only / A_0_+S_0_  (m/s)** | **Δ *c_s_*_,eff_  (%)** | ***ν*_eff_**  **A_0_-only / A_0_+S_0_** | **Δ *ν*_eff_**  **(%)** | ***E*_eff_ A_0_-only / A_0_+S_0_ (GPa)** | **Δ *E*_eff_  (%)** |
| --- | --- | --- | --- | --- | --- | --- | --- |
| 0° | 300/88 | 1253.131 / 1250.875 | -0.180 | 0.350 / 0.352 | 0.520 | 4.452 / 4.442 | -0.225 |
| 30° | 300/78 | 1197.354 / 1189.930 | -0.620 | 0.337 / 0.340 | 0.780 | 4.026 / 3.984 | -1.042 |
| 60° | 300/83 | 1176.542 / 1177.836 | 0.110 | 0.324 / 0.324 | 0.040 | 3.848 / 3.857 | 0.230 |
| 90° | 300/76 | 1105.529 / 1115.037 | 0.860 | 0.309 / 0.304 | -1.550 | 3.359 / 3.405 | 1.355 |
| 120° | 300/80 | 1154.953 / 1161.190 | 0.540 | 0.313 / 0.309 | -1.220 | 3.677 / 3.707 | 0.789 |
| 150° | 300/79 | 1182.440 / 1197.930 | 1.310 | 0.325 / 0.319 | -1.850 | 3.890 / 3.975 | 2.171 |
| 180° | 300/84 | 1248.522 / 1239.283 | -0.740 | 0.341 / 0.342 | 0.450 | 4.388 / 4.328 | -1.362 |
| 210° | 300/83 | 1247.958 / 1243.840 | -0.330 | 0.324 / 0.327 | 1.080 | 4.329 / 4.311 | -0.397 |
| 240° | 300/78 | 1159.884 / 1172.063 | 1.050 | 0.307 / 0.304 | -0.960 | 3.692 / 3.762 | 1.881 |
| 270° | 300/76 | 1134.515 / 1133.494 | -0.090 | 0.301 / 0.301 | -0.120 | 3.518 / 3.510 | -0.208 |
| 300° | 300/76 | 1122.345 / 1133.232 | 0.970 | 0.323 / 0.319 | -1.370 | 3.501 / 3.557 | 1.608 |
| 330° | 300/86 | 1224.650 / 1221.221 | -0.280 | 0.332 / 0.333 | 0.410 | 4.194 / 4.175 | -0.458 |

**Table S4.** S0-mode inclusion sensitivity for ROI B-front.

| **Direction** | **N (A_0_/S_0_)** | ***c_s_*_,eff_**  **A_0_-only / A_0_+S_0_  (m/s)** | **Δ *c_s_*_,eff_  (%)** | ***ν*_eff_**  **A_0_-only / A_0_+S_0_** | **Δ *ν*_eff_**  **(%)** | ***E*_eff_ A_0_-only / A_0_+S_0_ (GPa)** | **Δ *E*_eff_  (%)** |
| --- | --- | --- | --- | --- | --- | --- | --- |
| 0° | 300/90 | 1248.477 / 1249.601 | 0.090 | 0.332 / 0.335 | 0.830 | 4.361 / 4.378 | 0.387 |
| 30° | 300/82 | 1233.577 / 1230.246 | -0.270 | 0.329 / 0.330 | 0.420 | 4.246 / 4.227 | -0.436 |
| 60° | 300/78 | 1163.674 / 1161.230 | -0.210 | 0.300 / 0.301 | 0.310 | 3.697 / 3.684 | -0.348 |
| 90° | 300/77 | 1160.923 / 1175.899 | 1.290 | 0.297 / 0.291 | -2.050 | 3.671 / 3.749 | 2.115 |
| 120° | 300/79 | 1159.692 / 1170.593 | 0.940 | 0.314 / 0.311 | -0.820 | 3.710 / 3.773 | 1.689 |
| 150° | 300/83 | 1234.026 / 1244.145 | 0.820 | 0.315 / 0.311 | -1.280 | 4.205 / 4.262 | 1.335 |
| 180° | 300/89 | 1254.553 / 1250.664 | -0.310 | 0.330 / 0.333 | 0.810 | 4.397 / 4.379 | -0.419 |
| 210° | 300/80 | 1261.208 / 1251.371 | -0.780 | 0.316 / 0.318 | 0.610 | 4.396 / 4.334 | -1.410 |
| 240° | 300/85 | 1203.070 / 1210.649 | 0.630 | 0.304 / 0.300 | -1.460 | 3.965 / 4.001 | 0.919 |
| 270° | 300/72 | 1161.285 / 1163.143 | 0.160 | 0.289 / 0.289 | 0.100 | 3.650 / 3.662 | 0.343 |
| 300° | 300/78 | 1159.524 / 1176.105 | 1.430 | 0.321 / 0.315 | -1.960 | 3.731 / 3.820 | 2.390 |
| 330° | 300/83 | 1219.377 / 1219.987 | 0.050 | 0.324 / 0.324 | 0.120 | 4.133 / 4.138 | 0.129 |

**Note for Tables S2-S4:** Across all three scan datasets, conditional S_0_ inclusion changed $c_{s,\mathrm{eff}}$by at most 1.43%, $E_{\mathrm{eff}}$ by at most 2.39%, and the maximum absolute change in $\nu_{\mathrm{eff}}$ was approximately 0.006.

**Table S5.** Direction-dependent shear-wave velocity $c_{s,\mathrm{eff}}$ identified from the three analyzed scan datasets of the additively manufactured thermoset composite.

| **Direction** | **ROI A-front  *c_s,_*_eff_ (m/s)** | **ROI A-back**  ***c_s,_*_eff_ (m/s)** | **ROI B-front  *c_s,_*_eff_ (m/s)** | ***c_s,_*_eff_ Mean ± SD (m/s)** | **CV (%)** |
| --- | --- | --- | --- | --- | --- |
| 0° | 1320.934 | 1250.875 | 1249.601 | 1273.803 ± 40.821 | 3.205 |
| 30° | 1244.907 | 1189.93 | 1230.246 | 1221.694 ± 28.469 | 2.33 |
| 60° | 1219.351 | 1177.836 | 1161.23 | 1186.139 ± 29.937 | 2.524 |
| 90° | 1154.08 | 1115.037 | 1175.899 | 1148.339 ± 30.835 | 2.685 |
| 120° | 1194.099 | 1161.19 | 1170.593 | 1175.294 ± 16.951 | 1.442 |
| 150° | 1206.443 | 1197.93 | 1244.145 | 1216.173 ± 24.596 | 2.022 |
| 180° | 1303.776 | 1239.283 | 1250.664 | 1264.574 ± 34.423 | 2.722 |
| 210° | 1270.198 | 1243.84 | 1251.371 | 1255.136 ± 13.576 | 1.082 |
| 240° | 1234.766 | 1172.063 | 1210.649 | 1205.826 ± 31.629 | 2.623 |
| 270° | 1124.371 | 1133.494 | 1163.143 | 1140.336 ± 20.271 | 1.778 |
| 300° | 1166.836 | 1133.232 | 1176.105 | 1158.724 ± 22.558 | 1.947 |
| 330° | 1259.461 | 1221.221 | 1219.987 | 1233.556 ± 22.443 | 1.819 |

**Table S6.** Direction-dependent Poisson’s ratio $\nu_{\mathrm{eff}}$ identified from the three analyzed scan datasets of the additively manufactured thermoset composite.

| **Direction** | **ROI A-front *ν*_eff_** | **ROI A-back** ***ν*_eff_** | **ROI B-front *ν*_eff_** | ***ν*_eff_ Mean ± SD** | **CV (%)** |
| --- | --- | --- | --- | --- | --- |
| 0° | 0.360 | 0.352 | 0.335 | 0.349 ± 0.013 | 3.658 |
| 30° | 0.353 | 0.340 | 0.330 | 0.341 ± 0.012 | 3.382 |
| 60° | 0.335 | 0.324 | 0.301 | 0.320 ± 0.017 | 5.422 |
| 90° | 0.311 | 0.304 | 0.291 | 0.302 ± 0.010 | 3.361 |
| 120° | 0.319 | 0.309 | 0.311 | 0.313 ± 0.005 | 1.691 |
| 150° | 0.336 | 0.319 | 0.311 | 0.322 ± 0.013 | 3.965 |
| 180° | 0.357 | 0.342 | 0.333 | 0.344 ± 0.012 | 3.525 |
| 210° | 0.333 | 0.327 | 0.318 | 0.326 ± 0.008 | 2.316 |
| 240° | 0.320 | 0.304 | 0.300 | 0.308 ± 0.011 | 3.436 |
| 270° | 0.310 | 0.301 | 0.289 | 0.300 ± 0.011 | 3.512 |
| 300° | 0.329 | 0.319 | 0.315 | 0.321 ± 0.007 | 2.246 |
| 330° | 0.351 | 0.333 | 0.324 | 0.336 ± 0.014 | 4.092 |

**Table S7.** Direction-dependent effective modulus *E*_eff_ identified from the three analyzed scan datasets of the additively manufactured thermoset composite.

| **Direction** | **ROI A-front *E*_eff_ (GPa)** | **ROI A-back *E*_eff_ (GPa)** | **ROI B-front *E*_eff_ (GPa)** | ***E*_eff_ Mean ± SD  (GPa)** | **CV  (%)** |
| --- | --- | --- | --- | --- | --- |
| 0° | 4.983 | 4.442 | 4.378 | 4.601 ± 0.333 | 7.228 |
| 30° | 4.403 | 3.984 | 4.227 | 4.205 ± 0.210 | 5.003 |
| 60° | 4.168 | 3.857 | 3.684 | 3.903 ± 0.245 | 6.286 |
| 90° | 3.667 | 3.405 | 3.749 | 3.607 ± 0.180 | 4.983 |
| 120° | 3.950 | 3.707 | 3.773 | 3.810 ± 0.126 | 3.298 |
| 150° | 4.084 | 3.975 | 4.262 | 4.107 ± 0.145 | 3.523 |
| 180° | 4.844 | 4.328 | 4.379 | 4.517 ± 0.284 | 6.296 |
| 210° | 4.516 | 4.311 | 4.334 | 4.387 ± 0.112 | 2.561 |
| 240° | 4.226 | 3.762 | 4.001 | 3.996 ± 0.232 | 5.812 |
| 270° | 3.478 | 3.510 | 3.662 | 3.550 ± 0.098 | 2.772 |
| 300° | 3.800 | 3.557 | 3.820 | 3.726 ± 0.146 | 3.924 |
| 330° | 4.500 | 4.175 | 4.138 | 4.271 ± 0.199 | 4.667 |

**Table S8.** Initial material parameters and Levenberg-Marquardt regression settings used for dispersion-curve fitting.

| **Category** | **Parameters** | | **Initial value / Setting** | |
| --- | --- | --- | --- | --- |
| Initial Material Parameters | Shear wave velocity *c_s_* (m/s) | Aluminum | 3150.000 | bounds [2800, 3400] |
|  |  | Composite | 1250.000 | bounds [950, 1550] |
|  | Poisson’s ratio *ν* | Aluminum | 0.33 | bounds [0.30, 0.35] |
|  |  | Composite | 0.30 | bounds [0.20, 0.40] |
| Control Parameters | Convergence criterion | | 1 × 10⁻⁶ | |
|  | Maximum iterations *N_max_* | | 2000 | |
|  | Initial damping $\lambda_{0}$ | | MATLAB default | |
| Multistart Check | Initial parameter pairs | | 5×5 evenly spaced grid  *c_s_* ∈ [1050,1450] m/s  ν ∈ [0.22,0.38] | |
